# Food Swamps and Transportation Access: Intersecting Structural Determinants of Food Shopping and Access in Marginalized Urban Communities

**DOI:** 10.3390/ijerph22101481

**Published:** 2025-09-25

**Authors:** Summaya Abdul Razak, Abiodun T. Atoloye, Curtis Jalen Antrum, Kritee Niroula, Richard Bannor, Snehaa Ray, Emil Coman, Tania Huedo-Medina, Valerie B. Duffy, Kristen Cooksey Stowers

**Affiliations:** 1 Department of Allied Health Sciences, University of Connecticut, Storrs, CT 06269, USA; summaya.abdul_razak@uconn.edu (S.A.R.); cantrum@chdi.org (C.J.A.); ubl24004@uconn.edu (K.N.); richard.bannor@uconn.edu (R.B.); tania.huedo@ehu.eus (T.H.-M.); valerie.duffy@uconn.edu (V.B.D.); 2 Department of Nutrition, Dietetics, and Food Sciences, Utah State University, Logan, UT 84322, USA; abiodun.atoloye@usu.edu; 3 Child Health and Development Institute, Farmington, CT 06032, USA; 4 Department of Nutrition Sciences, University of Connecticut, Storrs, CT 06269, USA; snehaa.ray@uconn.edu; 5 Health Disparities Institute, University of Connecticut, Hartford, CT 06030-7030, USA; coman@uchc.edu; 6 Department of Clinical and Health Psychology and Research Methods, Faculty of Psychology, University of the Basque Country UPV/EHU, Barrio Sarriena s/n, 48940 Leioa Bizkaia, Spain; 7 Basque Foundation for Science, IKERBASQUE, 48011 Bilbao, Spain; 8 Rudd Center for Food Policy and Health, University of Connecticut, Hartford, CT 06103, USA

**Keywords:** food swamps, transportation access, food environment, health equity, dietary behavior, urban neighborhoods, social determinants of health, community-based participatory research

## Abstract

The study examined the relationship between food swamps and self-reported food shopping frequency and perceived food access, while considering transportation mode and travel time. This Community-Based Participatory Research study surveyed residents from six neighborhoods in Hartford. Individual-level food swamp exposure (the ratio of unhealthy to healthy food stores within a 0.5-mile radius of participants’ homes) was measured both objectively (using GIS-based methods) and subjectively (through self-reporting). Poisson regression models assessed the associations between food swamps and outcomes (shopping frequency by store types and perceived access to food), with transportation mode and travel time as moderators. Of 304 participants, 51% lived in subjective (n = 153) and 71% in objective (n = 198) food swamps. Food swamp exposure was associated with greater shopping frequency at unhealthy outlets (β = 0.12, *p* < 0.001), less access to healthier food (β = −0.13, *p* < 0.001), and increased access to unhealthy food (β = 0.08, *p* < 0.001). Transportation significantly moderates these relationships; bus riders reported the highest rates of unhealthy food purchasing (β = 0.17, *p* < 0.001). Longer travel times increased both healthy and unhealthy food access (β = 0.01, *p* < 0.001 for each). Food swamps interact with public transportation to contribute to food shopping and access, underscoring the need for integrated food and transportation policies to address structural barriers and promote health equity in underserved urban communities.

## 1. Introduction

Diet quality is associated with better outcomes and the prevention of nutrition-related diseases [1,2,3]. However, the diets of many U.S. adults are unhealthful, with persistent widening disparities [4,5,6,7]. Although data from the National Health and Nutrition Examination Survey find improvements in diet quality over the past decades, most US adults still report poor-quality diets that disproportionately affect low-income and racially marginalized communities [7,8]. A component of higher diet quality scores, greater fruit and vegetable intake, has been associated with greater neighborhood access to supermarkets and increased shopping frequency for healthy food [8,9]. Based on Solar and Irwin’s framework on the social Determinants of Health (SDOH), access to healthy food is not merely a matter of personal choice; upstream social, economic, and political conditions influence it. The SDOH framework suggests that health outcomes are driven by two interconnected layers: structural determinants, which include governance, public policies, and social factors such as material circumstances, physical environments, and behavioral aspects. This framework was expanded to highlight how the built environment, including neighborhood food environments, embeds these upstream forces into people’s daily lives and health trajectories [10].

Food swamps, defined as neighborhoods with a disproportionate number of unhealthy food outlets relative to healthier options, represent a distinct concept from food deserts and act as both intermediary and structural determinants within the SDOH framework, arising from historical and systemic inequities, and shape everyday exposure to the nutritional environment [11,12]. Unlike food deserts, which highlight the lack of healthy food retailers, food swamps focus on the excessive presence of unhealthy options that influence everyday nutritional exposure. Food swamps are especially common in racially segregated, under-resourced neighborhoods where zoning policies, commercial disinvestment, and targeted marketing have favored the growth of fast food and convenience stores over full-service supermarkets and culturally appropriate, health-promoting food outlets [13,14,15]. These patterns reflect broader systems of structural racism documented extensively in public health literature [16,17,18]. These environments influence food choices and shape dietary behaviors through structural differences in the availability, cost, and accessibility of healthy versus calorie-dense, high-fat food options, as well as targeted advertising [14,15,19].

Populations in food swamp areas are more likely to consume unhealthy food, and are further associated with poor health outcomes [14,20,21]. These environments promote the intake of energy-dense, nutrient-poor foods and beverages, contributing to higher rates of obesity, cardiovascular disease, type 2 diabetes, hypertension, as well as metabolic syndrome and increased stroke risk [20,22,23]. Exposure to food swamps also raises risks for other diet-related health issues, including metabolic dysfunction and cardiovascular risk factors. Importantly, exposure to food swamps is not random but aligned with broader systems of structural racism and economic exclusion, key mechanisms in the SDOH framework that sustain unequal health outcomes across different population groups [10]. Evidence indicates that low-income and racial/ethnic minority populations are more likely than high-income white populations to live in food swamps and, as a result, report higher rates of obesity and nutrition-related diseases, fueling disparities in diet and health outcomes among populations [13,24,25,26,27]. Black and Latinx communities experience disproportionate exposure to food swamps, with studies showing these populations are 2–3 times more likely to reside in such environments compared to white residents [13,28].

An essential theme in the US food research and policy is the reduction in nutrition-related health disparities [29]. One focus of these efforts is the elimination of food deserts. The conventional approach involves establishing full-service supermarkets in low-income and minority neighborhoods, thereby increasing access to healthy foods and, in turn, reducing the risk of obesity and diet-related chronic diseases [29,30]. However, establishing a new full-service supermarket in a low-income and low-access area may not lead to its utilization or influence the dietary intake of neighborhood residents [31]. Food shoppers shop at various locations, may frequently bypass the nearest retailers, and continue to access fast food outlets [32]. Thus, shopping frequency appears to be a key behavioral determinant of food access and diet quality. While some adults shop weekly or daily, others may shop only once or twice a month. These variations are influenced by household size, income, transportation access, and the nature of the neighborhood food environment [33,34,35]. More frequent grocery shopping at supermarkets is associated with healthier diets, while living in areas with higher exposure to fast food outlets correlates with increased consumption of unhealthy foods. Shopping frequency at different store types (healthy vs. unhealthy) and environmental exposure to food outlets represent distinct but related pathways through which structural and intermediary conditions, such as food swamps and transportation barriers, shape everyday health behaviors. While exposure creates opportunity, actual shopping frequency reflects realized behavior influenced by multiple factors including transportation access [32,36].

Transportation access fundamentally influences food shopping patterns and dietary choices. Travel mode mainly determines food selection and shopping frequency, with individuals who have personal cars having a broader range of shopping options outside food swamp areas and being less prone to regularly shop at unhealthy outlets [37,38]. Walking, biking, or relying on public transport may confine individuals to food options within their immediate vicinity, leading to more frequent shopping at local unhealthy outlets due to limited access to distant, healthier food choices. An important but often overlooked barrier for public transportation users is the physical limitation of carrying groceries. Fresh produce, dairy, and other healthy foods are often bulky, heavy, and perishable, making them difficult to transport on buses or while walking. This logistical constraint may push residents toward processed, packaged foods or prepared fast food that is easier to carry. This is particularly challenging for families with children or elderly household members who must manage both mobility assistance and grocery transport. These practical considerations underscore why transportation mode matters beyond simple access; it fundamentally shapes what types of food can feasibly be purchased and transported home. Travel time also plays a key role in shopping frequency, as those who must travel long distances to access stores, combined with limited time due to work or family obligations, may decrease their visits to healthy food stores or depend more on nearby convenience stores [39,40,41]. Essentially, transportation either restricts or expands food accessibility, shaping dietary and shopping habits [42,43]. This highlights the limitations of proximity-based interventions and underscores the need for more comprehensive strategies that consider spatial, economic, and behavioral factors.

Despite increasing recognition of food swamps as a public health issue, significant gaps still exist in the research literature. Few studies have explicitly explored how food swamps interact with transportation barriers to impact food shopping frequency and perceived access to healthy foods. While structural inequalities in food environments are well documented, less is understood about how both objective measures (e.g., store density and type) and subjective perceptions (e.g., perceived access) of food swamps influence actual shopping behaviors. The moderating effect of transportation and travel time remains largely unexplored, especially among historically marginalized urban populations.

The present study examines how food swamp exposure and transportation barriers interact to influence food shopping behaviors and perceived food access among residents in Hartford, Connecticut. Grounded in the SDOH framework and expanded to consider the environment, this study investigates both objective and subjective measures of neighborhood food swamp exposure in relation to individual-level shopping patterns.

The specific aims of the study are to:

1. Determine whether subjective and objective food swamp measures are associated with:
  - Self-reported food shopping frequency by store type.
  - Perceived food access by food category.
2. Assess whether the associations in Aim 1 vary based on:
  - Primary mode of transportation (e.g., car, bus, walk, rideshare).
  - Travel time to one’s regular grocery store.

By addressing these aims, the study seeks to generate evidence that can inform location-based interventions and urban food policies that are equity-driven, context-sensitive, and attentive to the lived experiences of structurally marginalized populations.

## 2. Materials and Methods

This study is part of a broader Community-Based Participatory Research (CBPR) collaboration in the federally designated North Hartford Promise Zone (NHPZ) focused on alleviating diet-related health inequities through neighborhood revitalization and dismantling food swamps and structural racism in the neighborhood environment. CBPR approach was employed based on extensive evidence demonstrating its effectiveness in addressing health disparities in marginalized communities [44,45]. CBPR is particularly appropriate for food environment research as it: (1) ensures cultural relevance of measures and interventions [46]. (2) builds community capacity for sustainable change [47]. and (3) addresses power imbalances inherent in traditional research approaches that have historically excluded community voices from food policy decisions [48]. This study approach aligns with successful CBPR food environment studies in Detroit [49] and Baltimore [50]. In line with the CBPR approach, the study design and data collection tools described below were co-developed with Community Principal Investigators (Community PIs) and a Community Advisory Board (CAB).

### 2.1. Study Setting

This study was conducted in a purposive sampling frame of 6 low-income, ethnically diverse neighborhoods in Hartford: Northeast, Upper Albany, Clay Arsenal, Asylum Hill, Frog Hollow, and Barry Square. In consultation with the Community PIs and the CAB, these sites were selected because most of the residents are Black and Latinx with low income. Importantly, there is variation across sites in terms of income, race/ethnicity, percentage of foreign-born residents, urbanicity, health risk factors, food retail environment, and NHPZ status. In 2015, three of the study sites (Northeast, Upper Albany, Clay Arsenal) were designated as Promise Zones under the Obama Administration as part of the U.S. Department of Housing and Urban Development’s Promise Zone initiative (US Department of Housing and Urban Development, 2015, 2020) [51]. Thus, this NHPZ designation reflects the high levels of disinvestment, high poverty, distress, unemployment, food insecurity, and crime in the community. For instance, the poverty rate in the NHPZ is 49% (compared to 33.6% in Hartford and 10.8% in CT) [52]. The NHPZ comprises 19 census block groups, and the comparison sites have 26 census block groups.

### 2.2. Participant Recruitment

**Figure 1:**
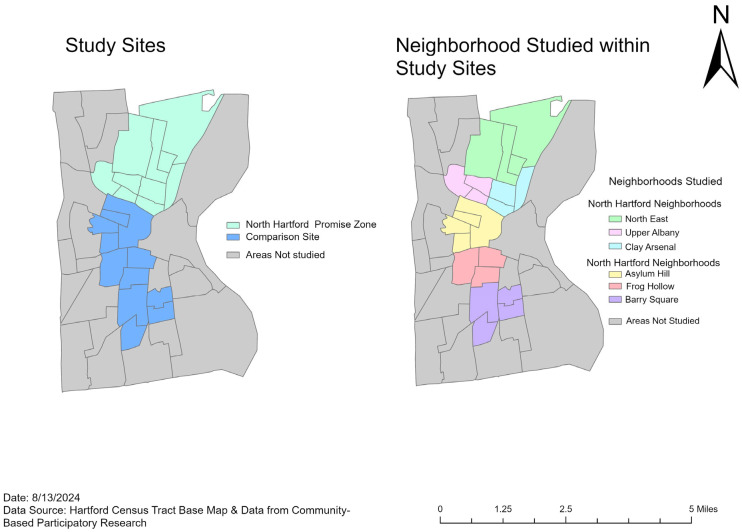
Geographic boundaries for the study sites and neighborhoods studied within the study sites. The left panel shows the North Hartford Promise Zone (light green) and Comparison Site (blue) areas. The right panel displays the specific neighborhoods studied within these sites, including Northeast, Upper Albany, and Clay Arsenal in the North Hartford Neighborhoods, and Asylum Hill, Frog Hollow, and Barry Square in the North Hartford Neighborhoods. This figure is an original visualization produced by the authors using Hartford Census Tract Base Map [53] & Survey Data from Community-Based Participatory Research.

**Figure 2:**
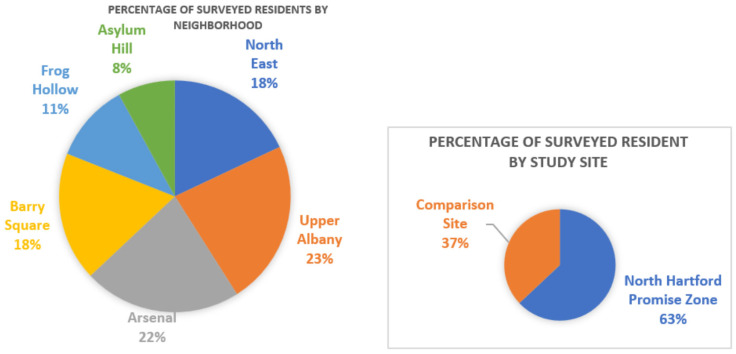
Percentage of surveyed residents by neighborhood and study site.

As illustrated in Figure 1 and Figure 2, participants were recruited through community events and flyers from six contiguous neighborhoods of North Hartford’s Promise Zone clusters (N = 192; green) and comparison clusters (N = 112; blue), with similar demographic and socioeconomic characteristics in Hartford. Comparison sites were included to establish generalizability across varying neighborhood food environments and demographic characteristics. Adults (≥18 years) were invited to participate in the study at each neighborhood if: (a) they resided in the North End of Hartford, or (b) any of the six designated neighborhoods.

### 2.3. Data Collection

Data collection occurred between August 2020 and May 2021, during the COVID-19 pandemic.

#### 2.3.1. Resident Survey Data

A resident survey was developed in REDCap and delivered electronically via telephone to 304 participants by trained research assistants and community residents trained as research partners, after obtaining written and informed consent.

Training and Quality Control: The data collection team underwent comprehensive training that included:

- Initial training on survey administration, ethics, and COVID-19 safety protocol;
- Role-playing and practice sessions;
- Ongoing supervision through weekly debriefing meetings.

Quality control measures included:

- Direct observation of 10% of survey administrations by the research coordinator;
- Daily data review for completeness and consistency;
- Regular team meetings to address challenges and ensure protocol fidelity.

To minimize bias, standardized scripts were implemented, rotated data collectors across neighborhoods, and conducted surveys at varied times to capture diverse residents.

Survey Instrument: The survey instrument gathered information on participants’ perception of their neighborhood food environment, focusing on the availability and total number of specific stores in their neighborhood. Participants chose from a “Yes” or “No” response to the availability of specific stores and indicated the total number of each store in their neighborhood. The survey assessed shopping frequency at different stores and the availability of specific foods in their neighborhood using validated questions from the Perceived Nutrition Environment Measures Survey (NEMS-P) by Green & Glanz [54]. Additional items and updated response options were developed through Community Advisory Board consultation and pilot testing to capture culturally specific foods and identify local transportation options. The survey also collected data on participants’ transportation to regular supermarkets and demographic information.

#### 2.3.2. Food Environment Audit Data

Pairs of trained research assistants collected primary data on food stores between December 2019 and January 2020 through an audit of the 6 neighborhoods’ food environments using a validated Food Swamp Environmental Assessment Tool (FS-EAT) [12]. The FS-EAT collects the street-level count of various food outlets within a neighborhood and assesses the store-level foods and beverages, surroundings, accessibility, and food promotions in each store. A total of 157 stores were audited, and the food store types audited included convenience stores with or without gas stations, national fast-food chain restaurants, supercenters, brown bag/carry-out food places, sit-down restaurants, food pantries, small and mid-size grocery stores, specialty stores, supermarkets, and discounted grocery stores.

### 2.4. Measures

All measures underwent cognitive testing with community members and were refined based on feedback before implementation.

Independent Variable: Food Swamp Index (FSI)

Food swamp exposure was measured at the individual level using both objective (GIS-based) and subjective (self-reported) data sources. Guided by prior literature on the health implications of different food outlet types [12]. Food stores were categorized as Traditional Fresh Food Retailers, Mixed Food Retailers, and Limited Fresh Food Retailers (Table 1).

Objective Food Swamp Index: Food store locations from the FS-EAT audit and resident survey data were geocoded into point data in ArcGIS Pro (version 10.4.1). The residents’ survey data points were then spatially joined to the Hartford census block group. The category of food store points from the audit data was then symbolized with basic colors and overlaid on the spatially joined Hartford base map. A 0.5-mile buffer was generated around each participant’s home address, and the number of food stores falling within each buffer, by category, was counted and assigned to each participant.

Subjective Food Swamp Index: Participants reported through the survey the number of specific food outlets (e.g., supermarkets, fast-food restaurants, dollar stores) located within approximately eight blocks of their homes. These self-reported counts were grouped into the same Traditional Fresh Food Retailers, Mixed Food Retailers, and Limited Fresh Food Retailers categories to mirror the objective classification.

Both objective and subjective FSI were calculated using the formula:

FSI = 100 × [(# of Traditional Fresh Food Retailers + # of Mixed Food Retailers, and Limited Fresh Food Retailers)/(# of total food outlets)].

This yielded individual-level FSI scores ranging from 0 to 100, where higher scores indicate greater exposure to unhealthy food outlets relative to healthy options. For analysis, individuals with FSI scores above the mean (subjective 70.2; Objective 66.1) were classified as residing in food swamp areas, while those with scores below the mean were classified as living in non-food swamp areas.

### 2.5. Dependent Variables

#### 2.5.1. Food Shopping Frequency

Participants were asked to think about all the food they buy or acquire for their family in a typical month and select how often they purchase food from each store type. Response options included “Every day” = 6, “Twice a week” = 5, “Once a week” = 4, “Twice a month” = 3, “Once a month” = 2, “Once every few months” = 1, and “Never” = 0. Sample statements included “How often do you shop at supermarkets for food?” For analysis, stores were grouped as Fresh Food Retailers, Mixed Food Retailers, and Limited Fresh Food Retailers using the meaningful grouping and weighting approach described by Song et al. [55] and Greco et al. [56]. Participants’ responses for store types within each category were summed to generate category scores. All participants missing responses to any store type (n = 47) were excluded from the summed scores. A composite score for shopping frequency at unhealthy stores was calculated as:

Limited Fresh Food Retail composite = [(sum of Limited Fresh Food Retail frequencies + sum of Mixed Food Retail frequencies)/(total shopping frequencies to food outlets)] × 100.

Composite scores ranged from 20 to 100 and were analyzed as a continuous variable, with higher values denoting more frequent shopping at unhealthy stores.

#### 2.5.2. Perceived Food Access

Participants indicated their level of agreement with statements about food availability, quality, and affordability in their neighborhood using a 5-point Likert scale: “Agree a lot” = 1; “Agree a little” = 2; “Neither Agree nor Disagree” = 3; “Disagree a little” = 4; and “Disagree a lot” = 5. The question was, “Please tell me how much you agree or disagree with each statement.” Sample statements included “Generally speaking, food from your culture is available in your neighborhood” and “Fresh fruits and vegetables are easy to get in your neighborhood.”

Food types were grouped as healthy (fruits and vegetables, dairy, whole grains, cultural food, and water) and unhealthy (junk food, fast food, and sugary drinks). Composite scores for the availability of each food category were generated by summing participants’ responses for all foods in each category. Participants missing responses for any food types were excluded from the respective composite (n = 21 for healthy foods, n = 10 for unhealthy foods). Composite scores for healthy food availability ranged from 0 to 33, and unhealthy food availability ranged from 0 to 12, with higher values denoting greater perceived availability.

### 2.6. Moderator Variables

#### Transportation Mode and Travel Time

Transportation mode and travel time were assessed as potential moderators based on theoretical frameworks suggesting these factors influence food access patterns and shopping behaviors. Participants indicated their primary means of travel to grocery stores from the following options: owning a car, car from carpool, car from family, borrowing a car from friends, taking a bus, walking, and other modes. Travel time to regular grocery stores was reported in minutes.

For analysis, transportation modes were collapsed into three categories: (1) owned car, (2) borrowed car (including carpool, family car, and borrowed from friends), and (3) bus and other modes (including bus, walking, and other transportation). Travel time was analyzed as a continuous variable.

### 2.7. Statistical Analysis

Descriptive statistics were computed to summarize participants’ sociodemographic characteristics, food shopping frequency, food availability, primary mode of travel, travel time to stores, and household composition. Means and standard deviations were calculated for continuous variables (e.g., age, shopping frequency, travel time, household size), while categorical variables (e.g., mode of travel, Food Swamp Index classification) were summarized using frequencies and percentages. Variable distributions were examined, and chi-squared tests were used to compare participants’ characteristics by food swamp classification. A Spearman correlation analysis was conducted to examine the association between primary mode of transportation and travel time, which indicated a significant relationship (r = 0.26, *p* < 0.0001), suggesting that reliance on shared or public transportation was linked with longer travel durations.

**Figure 3:**
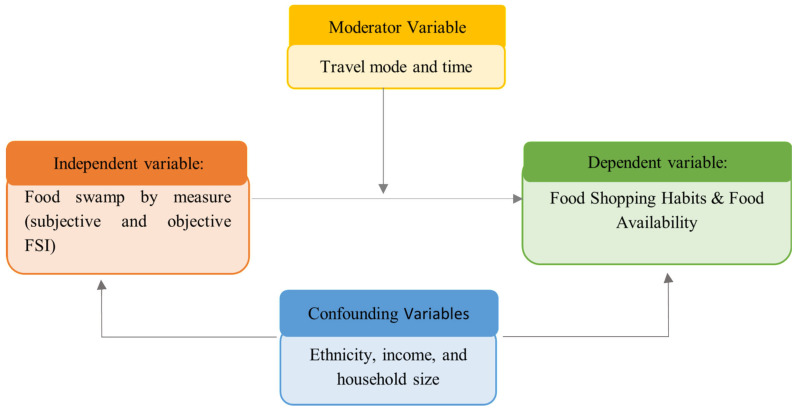
Conceptual Framework of the Regression Model.

Poisson regression models examined associations between individual-level Food Swamp Index exposure and three outcomes: (1) shopping frequency at unhealthy food outlets, (2) perceived access to healthy food, and (3) perceived access to unhealthy food. Separate models were estimated for objective and subjective Food Swamp Index scores. Additional models included interaction terms to test whether transportation mode and travel time moderate the relationship between food swamp exposure and outcomes. The framework for the regression model is illustrated in Figure 3.

Covariate Selection and Control: All regression models were adjusted for relevant covariates selected using the Disjunctive Cause Criterion approach [57,58,59], which allows variable selection based on theoretical knowledge of causal relationships with study variables. Existing literature suggests that income, ethnicity, and household size are associated with food access and food shopping habits [36,60,61,62,63]. Covariates controlled in the analysis included: Ethnicity (non-Latinx vs. Latinx), Annual household income (<$25,000 vs. ≥$25,000, and Household composition (number of family members aged 0–5 years, 6–17 years, and ≥18 years). Models also accounted for neighborhood clustering based on the two geographic strata (study and comparison sites). Because controlling for covariates can yield biased estimates of the impact of an independent variable on a dependent variable [64,65]. A conservative approach was taken by analyzing models with and without covariates.

Multiple Comparisons and Model Assessment: To address the risk of Type I error due to multiple comparisons across the six regression models, a Holm-Bonferroni correction was applied. Associations were considered statistically significant at adjusted *p*-value thresholds, with the most conservative threshold set at *p* < 0.0083.

Model fit was assessed using the iteration log, likelihood ratio chi-square, and model *p*-values. A lower final log-likelihood value, compared to the intercept-only model, was interpreted as evidence of improved model fit. The overall model was considered statistically significant at *p* < 0.05 (UCLA, 2021) [66]. All analyses were conducted using Stata^®^ version 17.0 SE Standard Edition (StataCorp, College Station, TX, USA).

## 3. Results

### 3.1. Descriptive Statistics

#### 3.1.1. Distribution Sociodemographic (Table 2)

Of the 304 participants recruited, all were included in the final analysis. Participants with missing data for specific variables were excluded only from the analysis requiring specific variables: shopping frequency composite scores (n = 47 excluded due to missing store frequency responses), healthy food access composite scores (n = 21 excluded due to missing food availability responses), and unhealthy food access composite scores (n = 10 excluded due to missing food availability responses).

A summary of participants’ sociodemographic, behavioral, and food environment characteristics is presented in Table 2. The sample was predominantly female (84%, n = 251) and Latinx (67%, n = 197), with a mean age of 47 years (median: 47; IQR: 18–88). Over half of the respondents (57%, n = 172) spoke English and had annual household incomes below $25,000 (71%, n = 207). Participants lived in households with an average of 0.4 children aged 0–5 years (range: 0–3 children), 0.9 children aged 6–17 years (range: 0–5 children), and 1.5 adults aged 18 years and above (range: 1–6 adults). Regarding food shopping behaviors, nearly all participants shopped at food stores located within Hartford (mean = 75.8%, median = 100%, IQR: 0–100%), while fewer shopped outside the city (mean = 24.1%, median = 0%, IQR: 0–100%). In terms of transportation, 48% (n = 137) of participants used their own car, 39% (n = 112) relied on carpools, family, or friends, and 12% (n = 35) used public transportation or other modes of transportation. The average travel time to a grocery store was 17.1 min (median: 12.5 min; IQR: 3-120 min).

**Figure 4:**
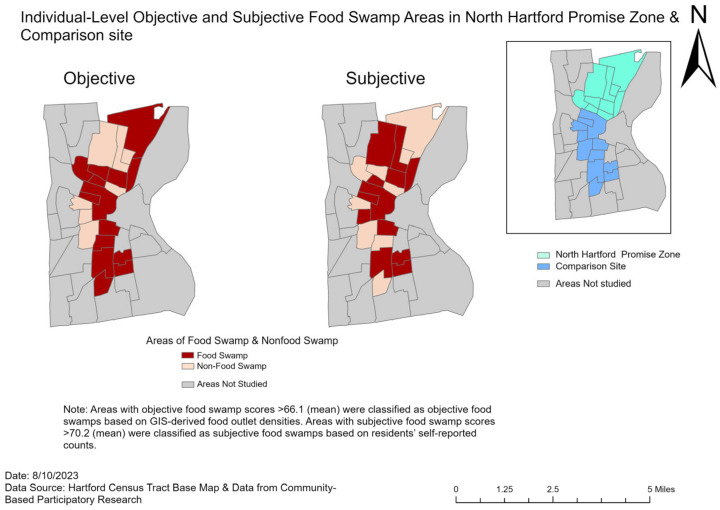
Individual Level Objective and Subjective Food Swamp Index in North Hartford Promise Zone (NHPZ) and comparison sites. This figure is an original visualization produced by the authors using Hartford Census Tract Base Map [53] & Survey Data from Community-Based Participatory Research.

Figure 4 shows areas with objective and subjective food swamps in North Hartford and comparison sites. Individual-level food swamp exposure was measured using both objective and subjective Food Swamp Index (FSI) scores. Based on objective FSI scores derived from GIS-based food store counts, 71.0% (n = 198) of participants were classified as living in food swamp areas. Based on subjective FSI scores from self-reported store counts, 51.3% (n = 153) of participants were classified as food swamp residents.

#### 3.1.2. Distribution of Demographic Characteristics by Food Swamp Status

Table 2 presents the distribution of participants’ demographic and behavioral characteristics stratified by both objective and subjective food swamp status. Significant age differences were observed across food swamp classifications. The mean age of participants was significantly higher among those classified as living in a food swamp area based on subjective FSI (49.4 vs. 44.4 years; *p* = 0.0019). A significant difference in age was also observed by objective FSI classification, with food swamp residents slightly younger than non-food swamp residents (46.9 vs. 47.1 years; *p* = 0.0019).

No statistically significant differences were observed in gender, ethnicity, or income across food swamp classifications, although a marginal difference in income was observed for objective FSI (*p* = 0.051). Language preferences differed significantly by objective food swamp status, with participants in objective food swamp areas more likely to speak English (76.3%) compared to those in non-food swamp areas (64.2%, *p* = 0.028). This difference was not statistically significant for subjective classification (*p* = 0.145).

Transportation patterns did not differ significantly across food swamp classifications. No significant differences were found in the primary mode of transportation to grocery stores or average travel time, regardless of food swamp status or classification type. Shopping location patterns also did not significantly differ across objective or subjective food swamp groups, with most participants reporting that they primarily shopped within Hartford.

#### 3.1.3. Distribution of Shopping Frequency, Healthy Food Access, and Unhealthy Food Access

**Figure 5:**
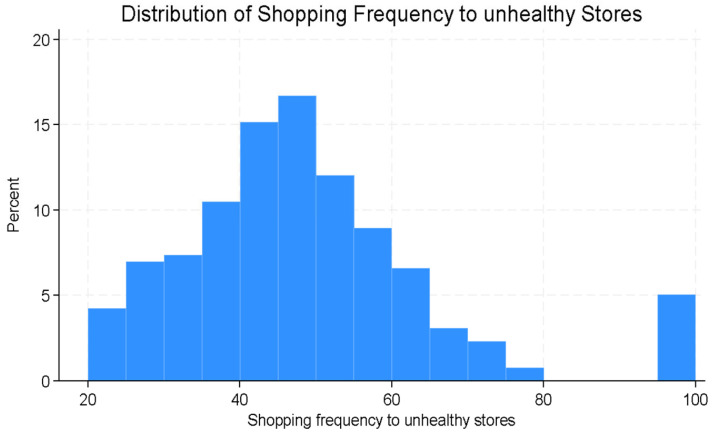
Distribution of shopping frequency to unhealthy stores. Note: Composite scores range from 20 to 100, representing frequency of shopping at outlets with limited fresh food options.

**Figure 6:**
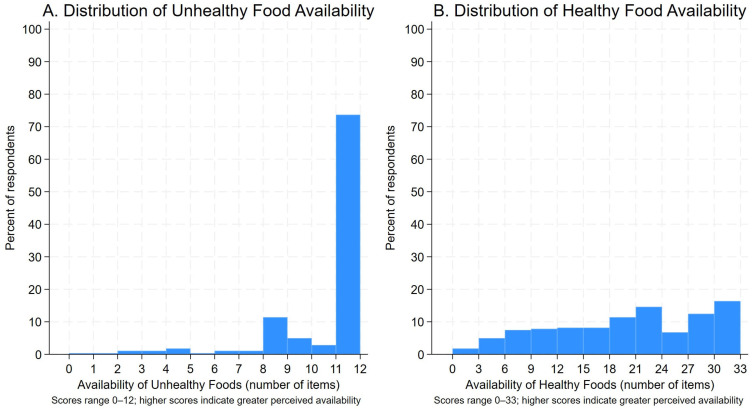
Distribution of unhealthy food availability and healthy food availability. Scores range from 0–12 for unhealthy foods and 0–33 for healthy foods; higher scores indicate greater perceived availability.

The distribution of the three main study outcomes (shopping frequency at unhealthy stores, perceived healthy food access, and perceived unhealthy food access) is presented in Figure 5 and Figure 6. The composite scores for shopping frequency at unhealthy stores ranged between 20 and 100 (mean [SD]., 48.4 [16.9].). Composite scores for perceived healthy food availability ranged from 0 to 33 (mean [SD]., 19.7 [8.8].) and perceived unhealthy food availability ranged from 0 to 12 (mean [SD]., 10.7 [2.3].).

### 3.2. Regression Results

#### Associations Between Food Swamp Exposure and Food Access Outcomes

Results from Poisson regression analyses examining associations between Food Swamp Index (FSI) exposure and food access outcomes are presented in Table 3. Five of the six tested associations remained statistically significant following Holm-Bonferroni correction.

Subjective Food Swamp Index Associations:

Subjective FSI was positively associated with shopping frequency at unhealthy food outlets (β = 0.12, 95% CI: 0.09 to 0.14, *p* < 0.001), negatively associated with perceived access to healthy food (β = −0.13, 95% CI: −0.19 to −0.07, *p* < 0.001), and positively associated with perceived access to unhealthy food (β = 0.08, 95% CI: 0.05 to 0.11, *p* < 0.001).

Objective Food Swamp Index Associations:

Objective FSI was positively associated with perceived access to unhealthy food (β = 0.06, 95% CI: 0.04 to 0.09, *p* < 0.001) and positively associated with shopping frequency at unhealthy food outlets (β = 0.01, 95% CI: 0.002 to 0.02, *p* = 0.017). The association between objective FSI and perceived access to healthy food was not statistically significant (β = −0.02, 95% CI: −0.04 to 0.001, *p* = 0.057).

### 3.3. Travel Mode and Travel Time Moderation of Food Swamp Associations with Shopping Frequency at Unhealthy Food Outlets

Results from analyses examining transportation moderation effects are presented in Table 4 and Table 5.

#### 3.3.1. Travel Mode Moderation

For objective food swamp models, significant interaction effects were observed between food swamp exposure and carpool/family/friend transportation (β = 0.09, *p* = 0.048) and between food swamp exposure and bus/other public transport (β = 0.09, *p* = 0.001). For subjective food swamp models, a significant interaction was observed between food swamp exposure and bus/public transport use (β = 0.17, *p* < 0.001). No significant interaction was observed for carpool/family transport in the subjective model (β = 0.02, *p* = 0.659).

#### 3.3.2. Travel Time Moderation

Travel time significantly moderated the association between food swamp exposure and unhealthy food shopping in the objective model. A positive interaction was observed between objective food swamp exposure and travel time (β = 0.004, *p* < 0.001). For subjective exposure, the crude model showed a positive interaction (β = −0.003, *p* < 0.001), but the adjusted model showed no significant moderation by travel time (β = −0.002, *p* = 0.192).

**Table 4:** Association between food swamp and unhealthy food access moderated by travel mode and time.

Outcome	Predictor Food Swamp by Measure	Objective				Subjective			
		Crude Coef.	*p*-Value	Adjusted Coef.	*p*-Value	Crude Model Coef.	*p*-Value	Adjusted Model Coef.	*p*-Value
Shopping Frequency to Unhealthy Stores	FSI main effect	−0.09	0.000	−0.09	0.000 *	0.08	0.117	0.09	0.018 *
	Travel mode main effect								
	Own car	-	-	-	-	-	-	-	-
	Car from others	−0.06	0.114	−0.05	0.006 *	−0.01	0.819	−0.003	0.768
	Bus and others	−0.07	0.086	−0.07	0.286	−0.04	0.008	−0.03	0.000 *
	Interaction								
	Food swamp|others car	0.09	0.048	0.10	0.260	0.01	0.852	0.02	0.659
	Food swamp|Bus and others	0.09	0.000	0.16	0.000 *	0.18	0.000	0.17	0.000 *
	FSI main effect	−0.11	0.000	−0.09	0.000 *	0.16	0.000	0.15	0.000 *
	Travel time main effect	−0.01	0.000	−0.01	0.000 *	−0.001	0.159	−0.002	0.288
	Food swamp|Travel time	0.01	0.000	0.004	0.000 *	−0.003	0.000	−0.002	0.192

* Statistically significant after Holm-Bonferroni correction for multiple comparisons (*p* < 0.0083).

### 3.4. Travel Mode and Time Moderation of Food Swamp Associations with Food Access

#### 3.4.1. Healthy Food Access

Both objective and subjective food swamp exposure were negatively associated with perceived healthy food access (adjusted β = −0.12 and −0.23, respectively; *p* < 0.001). Significant interactions were observed between food swamp exposure and carpool/family/friend transportation for both objective (β = 0.29, *p* < 0.001) and subjective models (β = 0.26, *p* < 0.001). Public transportation did not significantly moderate this relationship in either model.

Travel time moderated the association between food swamps and healthy food access. Positive interactions between food swamp exposure and travel time were observed for both objective (β = 0.01, *p* < 0.001) and subjective models (β = 0.01, *p* < 0.001).

#### 3.4.2. Unhealthy Food Access

Subjective food swamp exposure was positively associated with perceived access to unhealthy food (adjusted β = 0.04). A significant interaction was observed between subjective food swamp exposure and public transportation use (β = 0.08, *p* = 0.009). Negative interactions were observed between food swamp exposure and carpool/family/friend transportation for both objective (β = −0.12, *p* = 0.006) and subjective models (β = −0.10, *p* = 0.009).

Travel time significantly moderated the relationship between objective food swamp exposure and perceived access to unhealthy foods (interaction β = 0.01, *p* < 0.001).

**Table 5:** Association between food swamp and healthy food access moderated by travel mode at the individual level.

Outcome	Predictor Food Swamp by Measure	Objective				Subjective			
		Crude OR	*p*-Value	Adjusted OR	*p*-Value	Crude Model OR	*p*-Value	Adjusted Model OR	*p*-Value
Healthy Food Availability	FSI main effect	−0.12	0.000	−0.12	0.000 *	−0.21	0.000	−0.23	0.000 *
	Travel mode main effect								
	Own car	-	-	-	-	-	-	-	-
	Car from others	−0.28	0.000	−0.27	0.000 *	−0.18	0.000	−0.24	0.000 *
	Bus and others	0.07	0.724	0.05	0.775	0.12	0.012	0.10	0.195
	Interaction								
	Food swamp|others car	0.31	0.000	0.29	0.000 *	0.21	0.000	0.26	0.000 *
	Food swamp|Bus and others	0.02	0.934	0.06	0.847	−0.11	0.514	−0.10	0.416
	FSI main effect	−0.11	0.023	−0.08	0.055	−0.11	0.137	−0.13	0.079
	Travel time main effect	−0.02	0.000	−0.02	0.000 *	−0.01	0.000	−0.01	0.000 *
	Food swamp|Travel time	0.01	0.000	0.01	0.000 *	−0.00	0.748	0.00	0.784
Unhealthy Food Availability	FSI main effect	0.08	0.007	0.07	0.007 *	0.04	0.000	0.04	0.063
	Travel mode main effect								
	Own car	-	-	-	-	-	-	-	-
	Car from others	−0.08	0.016	−0.09	0.028 *	−0.13	0.017	0.13	0.110
	Bus and others	0.08	0.000	0.07	0.000 *	−0.05	0.674	−0.03	0.731
	Interaction								
	Food swamp|others car	0.02	0.010	0.05	0.063	0.09	0.020	0.09	0.109
	Food swamp|Bus and others	−0.12	0.006	−0.10	0.009 *	0.08	0.581	0.03	0.731
	FSI main effect	0.01	0.201	−0.01	0.466	0.01	0.839	0.03	0.693
	Travel time main effect	−0.01	0.000	−0.01	0.000 *	−0.01	0.001	−0.01	0.151
	Food swamp|Travel time	0.01	0.000	0.01	0.000 *	0.01	0.097	0.004	0.329

* Statistically significant after Holm-Bonferroni correction for multiple comparisons (*p* < 0.0083).

## 4. Discussion

This study examined how food swamp exposure and transportation barriers interact to influence food shopping behaviors and perceived food access among residents in low-income, racially diverse urban neighborhoods [in Hartford, Connecticut]. The results demonstrate substantial variation in food swamp exposure across participants. Higher exposure was consistently associated with increased shopping frequency at unhealthy food outlets, reduced perceived access to healthy foods, and greater perceived access to unhealthy foods. These associations were significantly moderated by transportation mode and travel time, revealing important nuances in how structural barriers compound the disadvantages of the food environment. The findings provide compelling evidence that food swamps are significantly associated with unhealthy food shopping patterns and diminished access to healthy foods, with these relationships being particularly pronounced among individuals with limited transportation options and longer travel times to grocery stores. These specific findings, particularly that bus riders showed the highest rates of unhealthy food purchasing (β = 0.17, *p* < 0.001) and that 71% of participants lived in objective food swamps, directly inform our policy recommendations discussed below.

The finding that food swamp exposure predicts more frequent shopping at unhealthy food outlets among predominantly Black and Latinx residents aligns with previous research demonstrating that neighborhood food environments shape dietary behaviors through mechanisms of availability and accessibility [13,14,67]. Studies have shown that individuals living in environments with unhealthy food options are more likely to purchase energy-dense foods [68,69]. Research has shown that proximity to fast food outlets is associated with increased consumption of energy-dense foods and a higher body mass index among low-income populations, reinforcing the patterns observed in this study [70,71]. The shopping behaviors documented in this study align with behavioral economic theories, which emphasize how environmental cues and choice architecture, the way food options are presented and made available to consumers, shape decision-making processes [72]. Choice architecture refers to the design of environments that influence people’s choices without restricting their freedom to choose, including factors such as the placement, availability, and marketing of different food options [73,74]. Behavioral economics posits that individuals often make irrational choices due to cognitive biases and environmental factors, which can be strategically manipulated to promote more informed decision-making outcomes [75]. Consumers often exhibit biases like procrastination and unrealistic optimism, which can lead to suboptimal choices [75]. By altering the choice architecture, these biases can be mitigated, leading to improved decision-making outcomes. Various nudging strategies, such as priming, salience, and default options, have been shown to effectively promote healthier food purchases [74]. For instance, default nudges that set healthier options as the preset choice can significantly alter consumer behavior [74,76].

The prevalence of unhealthy food options in food swamp neighborhoods creates a “choice environment” that nudges residents toward less healthy options through convenience, affordability, and marketing saturation [22,23]. These patterns are particularly pronounced among Black and Latinx communities, who are disproportionately represented in food swamp areas. Research demonstrates that low-income and racial/ethnic minority populations are more likely than high-income white populations to live in food swamps and consequently report higher rates of obesity and nutrition-related diseases, including hypertension, cardiovascular disease, and type 2 diabetes, leading to disparities in diet and health outcomes [13,24,25,26,27].

These trends align with the Social Determinants of Health framework guiding this study, where food swamps serve as both intermediary and structural determinants, thereby embedding historical inequities into daily experiences. The concentration of unhealthy food outlets in these neighborhoods stems from decades of discriminatory zoning policies, commercial disinvestment, and targeted marketing that have systematically favored convenience stores and fast-food establishments over full-service supermarkets [77,78,79]. Such exposure is not distributed randomly but rather aligned with broader systems of structural racism and economic exclusion, core mechanisms in the Social Determinants of Health framework that perpetuate unequal health outcomes across population groups.

It is worth noting that we observed differences in food swamp classification between objective (71% of participants) and subjective (51% of participants) measures. While both measures showed similar patterns of association with our outcomes, this discordance raises important questions about how residents perceive their food environments versus objective geographic assessments.

### 4.1. Perceived Food Access Disparities

The finding that food swamp residents report significantly lower access to healthy foods while perceiving greater access to unhealthy options illuminates the subjective experience of living in structurally disadvantaged food environments among predominantly Black and Latinx communities. While increased access to unhealthy foods and decreased access to healthy foods may appear as two sides of the same coin, our data suggest they operate as distinct dimensions, creating a compound disadvantage. Residents may simultaneously experience abundant unhealthy options nearby while healthy options remain distant or inaccessible due to transportation barriers. This creates not just a simple trade-off but layered challenges requiring different intervention strategies, both reducing unhealthy food saturation and improving healthy food access. This dual pattern, reduced access to healthy food coupled with increased access to unhealthy food, suggests that food swamps create a “double burden” that simultaneously limits healthy options while amplifying exposure to foods associated with poor health outcomes for historically marginalized populations. These perceptual disparities align with research by Molitor & Kehl and Warner et al. [80,81], who found that residents of low-income and minority neighborhoods consistently report lower perceived availability and quality of healthy foods compared to residents of more affluent areas. The findings of this study extend this work by demonstrating that food swamp characteristics specifically contribute to these perceptual differences among Black and Latinx residents, beyond general neighborhood socioeconomic status. This is particularly significant given that Black and Latinx communities are disproportionately represented in food swamps, with our study finding that 71% of predominantly Black and Latinx participants lived in objective food swamps.

The dual burden observed is particularly concerning, given extensive research demonstrating that perceived food access influences actual food purchasing decisions and dietary behaviors [82,83]. This creates what Baum and Zimmerman et al. [84,85], describe as a “reinforcement cycle” where poor food environments shape perceptions, which in turn influence behaviors that may perpetuate unhealthy eating patterns among communities already facing multiple structural disadvantages [86,87]. Longitudinal evidence supports how these perceptual and behavioral patterns interact over time to influence diet quality and health outcomes in low-income communities of color [88,89,90]. The subjective experience of food access is particularly important for understanding how structural inequities manifest in daily life for Black and Latinx families. When residents perceive limited access to healthy foods and abundant access to unhealthy options, these perceptions can influence shopping decisions even when healthy options may be technically available. This subjective dimension of food access represents an understudied aspect of how food environments perpetuate health disparities in communities that have historically faced systematic disinvestment and discriminatory policies.

### 4.2. Transportation as a Critical Moderator

#### 4.2.1. Travel Mode Effects

One of the most significant contributions of this study is demonstrating how transportation mode moderates food swamp effects among predominantly Black and Latinx residents, with individuals relying on public transportation or borrowed vehicles experiencing stronger negative associations between food swamp exposure and food access outcomes. Our findings revealed that residents residing in food swamps and relying on shared or public transportation were associated with increased frequency of shopping at unhealthy food retailers. In subjective food swamp areas, residents using public transportation showed the strongest association with unhealthy food shopping frequency. Conversely, shared transportation through carpools, family, or friends appeared to expand access beyond immediate food swamp environments, with significant interactions observed for healthy food access in both objective and subjective models. This finding extends previous research by revealing that the type of transportation matters as much as having transportation access at all. The differential effects by transportation mode align with research by Fuller et al. and Widener and Shannon [38,91], who demonstrated that personal vehicle ownership provides significantly greater spatial access to food retailers compared to public transportation or walking. This is particularly evident in urban settings where car ownership correlates with increased fresh food intake and lower body mass index (BMI) [92]. Previous studies found that reliance on public transportation constrains food shopping to immediate neighborhood options, often limiting residents to smaller stores with higher prices and lower food quality [93,94,95,96]. The present findings contribute to this literature by showing how these transportation constraints interact specifically with food swamp characteristics to compound access barriers for Black and Latinx communities.

#### 4.2.2. Vehicle Access and Social Networks: Understanding the Nuance

The findings regarding borrowed vehicles and carpools reveal important nuances about how social capital and community resources can buffer food environment disadvantages. When residents have access to vehicles through family members, friends, or informal carpool arrangements, they can escape the geographical constraints of their immediate food environment. This vehicle access through social networks operates differently from individual car ownership because it often involves coordinated shopping trips, potentially leading to more strategic food purchasing decisions. Family members or friends with vehicles may take residents to preferred stores outside their neighborhoods, potentially to larger supermarkets with better selection and prices, or to culturally appropriate food outlets that may not be available locally. However, this borrowed vehicle access also comes with constraints not present with personal vehicle ownership. Residents must coordinate their shopping schedules with others, may have limited time for shopping, and may need to compromise on store choices based on others’ preferences or destinations. Despite these limitations, our findings suggest that even this constrained vehicle access provides significant protective effects against food swamp disadvantages, highlighting the critical importance of social capital and community networks in food access.

The protective effect of personal vehicle ownership observed in this study is consistent with studies by Drewnowski et al. and Gustafson et al. [82,97], who found that car ownership significantly moderated relationships between neighborhood food environments and dietary outcomes. Recent research has also shown that online grocery ordering can serve as an alternative strategy for residents with transportation limitations, particularly in low-income communities where it may support better diet quality [98]. Even limited vehicle access through social networks can help buffer food environment disadvantages, highlighting the importance of social capital and community resources documented by Dean and Sharkey [99].

### 4.3. Transportation and Structural Inequities

These transportation patterns reflect what Clifton terms “transportation poverty,” which disproportionately affects low-income households and communities of color [40,90]. Transportation poverty encompasses not only lack of personal vehicle access but also inadequate public transportation systems that fail to connect residents to diverse food retail options [100]. In many urban areas with high concentrations of Black and Latinx residents, public transportation systems may have limited routes to suburban areas where larger supermarkets are located, infrequent service that makes grocery shopping time-consuming, and schedules that may not align with store operating hours or residents’ work schedules.

The timing and frequency of public transportation emerge as critical factors that our study suggests warrant greater attention in food access research. Bus routes that serve predominantly Black and Latinx neighborhoods often have less frequent service, particularly in evenings and weekends when many residents might prefer to shop. Additionally, routes may not directly connect food swamp areas to neighborhoods with better food retail options, requiring multiple transfers that make grocery shopping particularly burdensome for families with children or elderly members.

These study findings provide empirical evidence of how transportation inequities interact with food environment characteristics to create compounding disadvantages for marginalized populations. Limited transportation options can exacerbate the negative impacts of living in food swamps, areas saturated with unhealthy food outlets, by restricting individuals’ ability to reach healthier food sources beyond their immediate neighborhoods. As a result, Black and Latinx populations may face layered challenges: not only are they surrounded by unhealthy food options, but they also lack the transportation means to escape these environments. This interaction between transportation and food access creates a cycle of disadvantage that reinforces health disparities across already vulnerable communities.

### 4.4. Travel Time Implications

The moderating effect of travel time reveals additional complexity in the relationships between food environments, particularly for residents of food swamp areas. This study found that longer travel times were associated with both increased access to healthy foods and, paradoxically, greater access to unhealthy foods among residents of food swamps. Specifically, individuals residing in objective food swamps who reported longer travel times had higher perceived access to both healthy foods and unhealthy foods, suggesting that mobility over longer distances may alleviate some access constraints imposed by unhealthy retail environments. However, extended mobility may also expose residents to a broader spectrum of unhealthy food options, possibly along transit corridors or near non-local destinations, as evidenced by positive associations between travel time and unhealthy food access. This dual pattern suggests that mobility, even when it requires significant time investment, can expand food options in both directions for residents of predominantly Black and Latinx neighborhoods.

For healthy food access, this finding aligns with research by Lo et al., Wainer et al., and Jiao et al. [35,101,102,103], which shows that willingness to travel longer distances can overcome neighborhood-level food access barriers. Residents of food-poor neighborhoods often travel substantial distances to access preferred shopping destinations, sometimes bypassing closer options in favor of stores with better selection, prices, or quality [32,104,105,106]. However, the simultaneous increase in access to unhealthy food, combined with longer travel times, may reflect what Kestens et al. [107], describe as “activity space exposure”: the idea that individuals encounter food opportunities throughout their daily travel patterns, not just in their residential neighborhoods. Research by Bulle Bueno et al., Zenk et al., and Hirsch et al. [108,109,110], as shown that commuting routes often expose individuals to numerous fast food and convenience food options, which may explain why longer travel times are correlated with greater access to unhealthy food among our participants. Longer commuting times reduce the time available for health-related activities, including meal preparation and physical activity, which can lead to increased consumption of convenience foods [37,111]. Shorter distances are associated with healthier behaviors. Oostenbach’s findings indicate that healthier behaviors are more prevalent in areas with higher walkability [111].

This pattern also aligns with time-geography approaches to food access that emphasize how individual mobility patterns and time constraints shape food environment exposures [112,113]. The work of Vallée et al. and Perchoux et al. [114,115], demonstrates that food access patterns are better understood by examining individual activity spaces rather than just residential neighborhoods, supporting our finding that travel time significantly moderates the effects of the food environment.

### 4.5. Strengths

This study has several notable strengths that enhance confidence in the findings. The Community-Based Participatory Research approach, conducted in partnership with Community Principal Investigators and a Community Advisory Board (CAB), ensures that research priorities and methods reflect community needs and perspectives, enhancing the validity and relevance of findings. The use of both objective (GIS-based) and subjective (self-reported) food swamp measures provides a more comprehensive assessment of food environment exposure than studies relying on single measurement approaches. The convergent findings across both measures strengthen confidence in the robustness of observed associations. The focus on transportation mode and travel time as moderators addresses a significant gap in food environment research. Most previous studies have treated transportation as a simple binary rather than examining how different transportation modes and travel constraints interact with food environments. Finally, the sample’s demographic characteristics, predominantly low-income Black and Latinx residents, represent populations disproportionately affected by food environment inequities but underrepresented in much food environment research.

### 4.6. Limitations

Several limitations should be considered when interpreting the findings. The cross-sectional design precludes causal inferences about relationships between food swamps, transportation, and food access outcomes. While the theoretical framework suggests that food environments influence behaviors, reverse causation or unmeasured variables could explain some observed associations. The reliance on self-reported measures for shopping frequency and perceived food access introduces potential reporting bias, although there is consistency of patterns across multiple measures. The data collection period coincided with the COVID-19 pandemic, which substantially altered food shopping behaviors, including increased reliance on delivery services, changes in store hours and capacity limits, and supply chain disruptions. The pandemic context may limit the generalizability of these findings to non-pandemic periods. However, the pandemic also highlighted and exacerbated existing food access inequities, making these findings particularly relevant for understanding vulnerabilities in the food system.

The categorization of food stores, while based on validated measures, may not fully capture the evolving nature of food retail. Dollar stores, traditionally categorized as sources of unhealthy food, are increasingly adapting to serve as primary food sources in low-income neighborhoods, with some locations now offering fresh produce and healthier options. Conversely, supermarkets in low-income areas may offer a limited variety of healthy foods compared to those in affluent neighborhoods. Future research should consider more nuanced store assessment methods that evaluate actual food availability and quality within individual stores rather than relying solely on store type.

The geographic focus on Hartford, Connecticut, may limit generalizability to other urban contexts with different food environments, transportation systems, or demographic compositions. Although key sociodemographic variables were controlled for, unmeasured confounders, such as food preferences, cultural factors, health conditions, or other neighborhood characteristics, could still influence the observed relationships.

### 4.7. Implications for Health Equity

#### Structural Racism and Food Justice

Given that our sample was predominantly Black and Latinx (67%) residents with low incomes, with 71% of participants living in objective food swamps and 51% in subjective food swamps, these findings highlight how structural inequities in food environments disproportionately impact historically marginalized communities. The findings provide quantitative evidence of how structural racism manifests in food environments and shapes health-related behaviors. The substantial proportion of the predominantly Black and Latinx sample living in food swamps reflects historical legacies of redlining, discriminatory zoning practices, and systematic disinvestment in communities of color [13,116,117,118,119,120]. The identified transportation barriers exacerbate these inequities by limiting residents’ access to healthier options outside their immediate neighborhoods. This intersection of food environment and transportation inequities creates what Pothukuchi [121], terms “compound food access barriers” for communities of color, consistent with research by Lee et al. and Shannon [93,122]. These patterns exemplify what Bullard [123], described as “environmental racism,” more recently applied to food environments by Morland and Filomena [123]. The systematic concentration of unhealthy food outlets combined with transportation barriers creates “racialized food landscapes” that perpetuate health disparities through environmental mechanisms rather than individual choices alone [124].

### 4.8. Policy Implications

#### 4.8.1. Beyond Food Desert Interventions

The results have important implications for food policy interventions that have traditionally focused on eliminating food deserts by establishing new supermarkets in underserved areas serving predominantly Black and Latinx communities. While increasing healthy food retail options remains important, our findings suggest such interventions may be insufficient without addressing broader food swamp contexts and transportation barriers that disproportionately affect these populations. The complex associations observed between objective food environments and access outcomes suggest that simply changing retail landscapes may not immediately translate to improved food access experiences for residents who face multiple, intersecting barriers.

This aligns with mixed evidence from supermarket intervention studies, including evaluations of the Pennsylvania Fresh Food Financing Initiative [31], and the Pittsburgh Hill District intervention [89], which showed limited impacts on dietary behaviors despite improved access metrics. Studies by Elbel et al. and Ghosh-Dastidar et al. [90,125], have similarly found that supermarket interventions alone produce modest changes in food purchasing patterns among low-income communities of color. These studies support arguments by Walker et al. and Gittelsohn et al. that effective food environment interventions must address the full retail ecosystem, including regulations on unhealthy food outlets, rather than focusing solely on adding healthy options [50,126].

These findings emphasize the need for comprehensive approaches integrating food retail development with transportation planning and community economic development, as advocated by Caspi et al. and Ver Ploeg et al. [83,95,127]. Research has also demonstrated that online grocery ordering can serve as an important strategy for addressing food access barriers in urban low-income communities, [98,128], particularly when residents face both food swamp environments and transportation constraints. Studies by Dean and Sharkey demonstrate that transportation constraints often prevent residents from utilizing new food retail options, even when geographically accessible [99].

#### 4.8.2. Multi-Level Intervention Approaches

The findings suggest that effective interventions to address food environment inequities must operate at multiple levels and address both food retail environments and transportation barriers simultaneously. Based on our findings among predominantly Black and Latinx residents, policy recommendations include:

Transportation Infrastructure: Given our findings that public transportation users in food swamps showed the strongest associations with unhealthy food shopping (β = 0.17, *p* < 0.001), targeted investments in public transportation infrastructure are essential. Specific recommendations include:

- Enhanced Bus Routes and Frequency: Our data showing longer travel times for public transit users justifies increasing service frequency to predominantly Black and Latinx neighborhoods.
- New routes should directly connect food swamp areas to neighborhoods with diverse food retail options, minimizing transfer requirements that burden families with children or elderly members.
- Subsidized Transportation Programs: The interaction between transportation mode and food swamp exposure supports implementing grocery-specific transportation vouchers, similar to programs that provide reduced-fare transportation to medical appointments. This could include partnerships with ride-sharing services or dedicated shuttle services to supermarkets.
- Mobile Food Markets: Supporting mobile food markets that bring healthy, affordable options directly to underserved areas, particularly those with limited public transportation access.

Nutritional Environment Interventions: Building on our findings regarding access to unhealthy foods, policies should address the broader nutritional environment:

- Sugar-Sweetened Beverage Taxes: Implementing taxes on sugar-sweetened beverages to reduce consumption and generate revenue for health promotion programs in affected communities. Revenue from SSB taxes should be reinvested in communities disproportionately affected by diet-related diseases.
- Water Access Infrastructure: Installing public drinking water fountains and refill stations in predominantly Black and Latinx neighborhoods to provide free access to healthy beverages and reduce reliance on sugar-sweetened alternatives. This is particularly important given our findings of greater perceived access to unhealthy beverages in food swamp areas.

Economic Development: Rather than simply adding supermarkets to food desert areas, economic development efforts should focus on creating diverse, community-controlled food retail ecosystems that include culturally appropriate options and address the full spectrum of food access barriers. This includes supporting Black and Latinx-owned food businesses, culturally specific food retailers, and cooperative grocery stores that serve community needs while building local economic capacity.

Zoning and Land Use Policy: With 71% of our predominantly Black and Latinx participants living in objective food swamps, municipalities should pursue a dual strategy that both limits oversaturation and improves existing stores:

- Limiting Oversaturation: Implement density restrictions on new fast food restaurants in areas already oversaturated with such establishments.
- Improving Existing Stores:
  ○ Given the evolving role of dollar stores, convenience stores, and corner stores as primary food sources in low-income neighborhoods, policies should incentivize healthy food stocking rather than limiting these essential community resources;
  ○ Healthy Food Financing Initiatives: Provide grants or low-interest loans to small store owners for refrigeration equipment to stock fresh produce, dairy, and other perishables.
- Tax Incentives: Offer tax credits to convenience and corner stores that dedicate a minimum percentage of shelf space to fresh fruits, vegetables, whole grains, and other healthy options.
- Wholesale Purchasing Cooperatives: Facilitate collective buying arrangements that allow small stores to purchase healthy foods at competitive prices.
- Technical Assistance Programs: Provide training on produce handling, storage, and marketing to help store owners successfully stock and sell fresh foods.
- Healthy Corner Store Certification Programs: Create voluntary certification programs that provide marketing benefits and customer recognition for stores meeting healthy food stocking standards.
- Zoning Bonuses: Allow expanded operating hours or additional square footage for stores that meet healthy food availability criteria.
- SNAP/WIC Authorization Support: Assist small stores in obtaining and maintaining authorization to accept food assistance benefits, particularly for fresh produce.
- Partnership Approaches:
  ○ Develop partnerships between small stores and local urban farms or food hubs to ensure a consistent fresh food supply;
  ○ Create “store-within-a-store” models where health-focused vendors can operate produce sections within existing convenience stores.

This dual approach recognizes that dollar stores and convenience stores are often the most accessible food sources for transit-dependent residents and should be improved rather than eliminated. The focus shifts from restricting access to transforming these existing resources into healthier options while preventing further oversaturation of fast food outlets.

#### Community-Centered Solutions

The CBPR approach used in this study highlights the importance of community leadership in developing food environment solutions. Effective interventions should build on existing community assets and address priorities identified by residents themselves. This includes supporting community gardens, food cooperatives, and other community-controlled food initiatives that address both food access and community economic development goals.

Online Food Access with SNAP: Given transportation barriers identified in our study, expanding online purchasing options for SNAP recipients represents an important policy opportunity. Online grocery shopping with SNAP benefits could help residents overcome transportation constraints while accessing healthier food options, with research showing that online grocery ordering can support higher diet quality among adults in low-income, low-access food environments when combined with food assistance program participation [84]. However, this requires ensuring adequate internet access and delivery services in predominantly Black and Latinx neighborhoods.

Community Asset Building: Rather than deficit-based approaches that focus solely on what communities lack, interventions should build on existing community strengths, social networks, and cultural food practices. This includes supporting community kitchens, food preservation programs, and initiatives that celebrate and promote culturally appropriate healthy foods within Black and Latinx communities.

### 4.9. Future Research Directions

- Future research should employ longitudinal designs to better establish causal relationships between changes in the food environment, transportation access, and food-related behaviors and health outcomes. Natural experiments around new food retail development, transportation infrastructure changes, or policy interventions could provide valuable causal evidence.
- Future research should also explore how emerging food access strategies, such as online grocery ordering with food assistance benefits, may help address the intersection of food swamp exposure and transportation barriers documented in this study. Research in similar urban contexts has shown promise for online food access interventions [87,115], suggesting this may be a viable complement to traditional food environment interventions. Replication of this research in diverse geographic contexts, including rural areas, different regions, and communities with varying demographic compositions, would enhance understanding of how food swamp effects vary across different settings and populations.
- Research examining the specific mechanisms through which food swamps influence food behaviors could inform more targeted interventions. This might include studies of food marketing and advertising, price comparisons across different food environments, or qualitative research on decision-making processes in different food environment contexts.
- Future research should also explore how digital innovations, including food waste reduction apps (e.g., Too Good To Go, Flashfood) and grocery delivery platforms, might mitigate or exacerbate food access inequities. These technologies could potentially provide alternative pathways to affordable food but may also introduce new barriers related to digital literacy, smartphone access, and payment methods. Understanding the intersection of digital and physical food access represents an important frontier for food justice research.

## 5. Conclusions

This study provides robust evidence that food swamps significantly impact food shopping behaviors and perceived food access among low-income Black and Latinx residents, with these effects being substantially moderated by transportation access. The findings highlight the inadequacy of traditional food desert frameworks that focus solely on the presence or absence of supermarkets without considering the broader food retail ecosystem and structural barriers that shape food access. The transportation disparities identified reveal how multiple structural inequities intersect to create compounding disadvantages for historically marginalized communities. The practical logistics of grocery shopping via public transportation, including the physical burden of carrying heavy, bulky fresh foods, represent an additional layer of disadvantage that may push residents toward processed or fast foods regardless of their preferences. These findings underscore the need for comprehensive, equity-focused interventions that address food environments, transportation infrastructure, and broader systems of structural racism simultaneously. Ultimately, achieving food justice requires moving beyond individual-level interventions to address the structural determinants that create and maintain food environment inequities. This research contributes to the broader agenda by providing evidence of how food swamps and transportation barriers interact to perpetuate health disparities, while pointing toward multi-level solutions that can create more equitable and health-promoting food environments for all communities.

## Figures and Tables

**Table 1:** Food store type by category.

Traditional Fresh Food Retailers	Mixed Food Retailers	Limited Fresh Food Retailers
Supermarket	Sit-down restaurant	Convenience store with gas
Mid-size grocery store	Take-Out restaurant	Convenience store without gas
Small-sized grocery store		Dollar store
Specialty store		Fast food restaurant
Community garden		Corner store
Home Garden		Discount store
Farmers market		
Food Pantry		
Supercenter		

Note: Store categorization represents general patterns; individual stores within categories may vary in their actual food offerings.

**Table 2:** Distribution of demographics by the food swamp (N = 304).

Characteristics	N (%)/M (SD)	Objective			Subjective		
		Food Swamp N = 198 (71%)	Non-Food Swamp N = 81 (29%)	*p*-Value	Food Swamp N = 153 (51.3%)	Non-Food Swamp N = 145 (48.7%)	*p*-Value
Age (M/SD)	46.8 (15.0)	46.9 (14.9)	47.1 (15.7)	0.0019	49.4 (15.4)	44.4 (14.1)	0.0019
Gender				0.183			0.196
Female	251 (84.0)	161 (70.0)	69 (30.0)		122 (48.6)	124 (50.4)	
Male	47 (15.7)	34 (77.3)	10 (22.7)		29 (61.7)	18 (38.3)	
Language				0.028			0.145
English	172 (56.6)	119 (76.3)	37 (23.7)		93 (55.0)	76 (45.0)	
Spanish	132 (43.42)	79 (64.2)	44 (35.8)		60 (46.5)	69 (53.5)	
Ethnicity				0.173			0.533
Not Latinx	98 (33.2)	66 (75.9)	21 (24.1)		52 (53.6)	45 (46.4)	
Latino	197 (66.8)	124 (67.8)	59 (32.2)		98 (49.8)	99 (50.3)	
Income				0.051			0.090
Below $25,000	207 (71.1)	127 (67.2)	62 (32.8)		100 (48.5)	106 (51.5)	
Above $25,000	84 (28.9)	61 (79.2)	16 (20.8)		50 (59.5)	34 (40.5)	
Household size (M/SD)							
Family members 0–5 years	0.4 (0.7)	0.4 (0.7)	0.3 (0.6)	0.7651	0.4 (0.6)	0.4 (0.7)	0.1208
Family members 6–17 years	0.9 (1.2)	0.8 (1.3)	0.9 (1.1)	0.9031	0.9 (1.1)	0.9 (1.4)	0.7121
Family members 18 and above	1.5 (1.1)	1.5 (1.1)	1.4 (1.2)	0.7016	1.6 (1.1)	1.4 (1.1)	0.1608
Travel mode to supermarket				0.804			0.471
Own car	137 (48.2)	89 (71.8)	35 (28.2)		75 (55.2)	61 (44.9)	
Car from family/friend/carpool	112 (39.4)	70 (68.0)	33 (32.0)		53 (47.3)	59 (52.7)	
Bus and other modes	35 (12.4)	23 (71.9)	9 (28.1)		18 (51.4)	17 (48.6)	
Travel time to store (M/SD)	17.1 (14.6)	17.6 (15.7)	15.5 (11.5)	0.862	17.8 (15.3)	16.4 (14.1)	0.792
Shopping location							
In Hartford (M/SD)	75.8% (32.8)	77.9 (32.4)	73.8 (33.2)	0.183	76.5 (31.0)	75.2 (34.5)	0.366
Outside Hartford (M/SD)	24.1% (32.6)	26.1 (32.9)	22.1 (32.4)	0.809	23.6 (40.0)	24.6 (34.2.9)	0.601

**Table 3:** Associations between individual-level food swamp exposure and food shopping frequency, healthy food access, and unhealthy food access (Poisson regression results).

Outcome	Predictor Food Swamp (by Measure)	Adjusted β (95% CI)	*p*-Value
Shopping Frequency to Unhealthy Food Outlets	Objective FSI	0.01 (0.002, 0.02)	0.017
	Subjective FSI	0.12 (0.09, 0.14) *	<0.001
Healthy Food Access	Objective FSI	−0.02 (−0.04, 0.001)	0.057
	Subjective FSI	−0.13 (−0.19, −0.07) *	<0.001
Unhealthy Food Access	Objective FSI	0.06 (0.04, 0.09) *	<0.001
	Subjective FSI	0.08 (0.05, 0.11) *	<0.001

* Statistically significant after Holm-Bonferroni correction for multiple comparisons (*p* < 0.0083).

## Data Availability

The data presented in this study are available on request from the corresponding author.

## Abbreviations

The following abbreviations are used in this manuscript:

SDOH	Social Determinants of Health
NHPZ	North Hartford Promise Zone
CBPR	Community-Based Participatory Research
CAB	Community Advisory Board
FS-EAT	Food Swamp Environmental Assessment Tool
FSI	Food Swamp Index
